# Hepatic Parenchymal Changes following Transcatheter Embolization and Chemoembolization in a Rabbit Tumor Model

**DOI:** 10.1371/journal.pone.0070757

**Published:** 2013-08-14

**Authors:** Yong Wang, Bin Xiong, Bin Liang, Hui Zhao, Hui Li, Jun Qian, Hui-Min Liang, Gan-Sheng Feng, Chuan-Sheng Zheng

**Affiliations:** Department of Radiology, Union Hospital, Tongji Medical College, Huazhong University of Science and Technology, Wuhan, P. R. China; Vanderbilt University Medical Center, United States of America

## Abstract

**Objective:**

To compare the effects of transcatheter arterial chemoembolization (TACE) with transcatheter arterial embolization (TAE) on liver function, hepatic damage, and hepatic fibrogenesis in a rabbit tumor model.

**Materials and Methods:**

Thirty-nine New Zealand white rabbits implanted with VX2 tumors in the left liver lobes were randomly divided into three groups: TAE, TACE, and control group. In the TAE group (n = 15), polyvinyl alcohol particles (PVAs) were used for left hepatic artery embolization. In the TACE group (n = 15), the tumors were treated with left hepatic arterial infusions of a suspension of 10-hydroxycamptothecin and lipiodol, followed by embolization with PVAs. In the control group (n = 9), the animals received sham treatment with distilled water. Serum and liver samples were collected at 6 hours, 3 days and 7 days after treatment. Liver damage was measured using a liver function test and histological analyses. Liver fibrogenesis and hepatic stellate cell (HSC) activation were evaluated using Sirius Red and anti-alpha-smooth muscle actin (α-SMA) immunohistochemical stains.

**Results:**

TACE caused liver injury with greater increases in serum alanine aminotransferase and aspartate aminotransferase levels on day 3 (P<0.05). Histological analyses revealed increased hepatic necrosis in adjacent non-tumorous liver tissue from day 3 compared to the TAE group (Suzuki score of 2.33±1.29 versus 1.13±1.18, P = 0.001). HSC activation and proliferation were significantly increased in the TACE group compared to the control group at 3 and 7 days after treatment (0.074±0.014 *vs.* 0.010±0.006, and 0.088±0.023 *vs.* 0.017±0.009, *P*<0.05). Sirius Red staining demonstrated a statistically significant increase in collagen deposition in the livers in the TACE group 7 days after embolization compared to the control group (0.118±0.012 *vs.* 0.060±0.017, *P* = 0.05).

**Conclusion:**

The results of this animal study revealed that TACE induced prominent hepatocellular damage and hepatic fibrogenesis, which compromised liver function and may be responsible for chronic liver decompensation.

## Introduction

Transcatheter arterial embolization (TAE) and chemoembolization (TACE) have been increasingly used as effective palliative treatments for unresectable hepatic tumors [1]–[3]. TAE and TACE induce ischemic or toxic injury both in the tumors and in the adjacent hepatic tissues, which sometimes results in hepatic failure. Recent studies have suggested that the damage to normal liver parenchyma may impair the therapeutic efficacy of TAE and TACE. Therefore, a thorough understanding of hepatic pathology following transarterial therapy may be helpful for the preservation of liver function.

There has been some speculation regarding the possible causes of TAE- and TACE-induced liver damage. Numerous studies have concentrated mainly on hepatocyte necrosis [4], [5], bile ductal or portal vein endothelial damage [6]–[8], and inflammatory reactions [9], [10]. Liver fibrogenesis, which may be considered to be a wound healing response to liver injury, is characterized by both a quantitative increase and a qualitative change in extracellular matrix (ECM) composition. During this process, hepatic stellate cells (HSCs) are activated and proliferate, contract, induce matrix degradation, and synthesize collagen. HSC activation and liver fibrogenesis play critical roles in the homeostasis of liver repair, regeneration and fibrosis after liver injury [11], [12].

Given the fact that ischemia and chemotherapeutic cytotoxicity are potent stimuli for liver repair, we hypothesized that TACE and TAE may induce hepatocyte necrosis, activate HSCs and initiate fibrogenesis, which are involved in the pathogenesis of hepatic fibrosis and are responsible for chronic liver decompensation. To test this hypothesis, the effects of TACE and TAE on embolized and unembolized liver tissues were examined using morphometric analyses in a rabbit model.

## Materials and Methods

### Animal Models and Experimental Design

Adult New Zealand white rabbits, weighing 3.0–3.5 kg, were used in this study. Experiments were performed in compliance with the Ethical Guidelines for the Care and Use of Laboratory Animals, as approved by the Animal Care Committee of Hubei Province, China. The animals were anesthetized with an intravenous injection of 30 mg/kg of sodium pentobarbital, and all efforts were made to minimize suffering.

Thirty-nine New Zealand white rabbits with VX2 tumors that had been transplanted into the livers were used in this study. VX2 tumors were implanted by embedding 1 mm tissue cubes below the left lobe capsule for 17–18 days. Each rabbit was then subjected to one of the following three treatment groups: (1) The TAE group (n = 15), in which the tumor bearing liver lobe was embolized using polyvinyl alcohol particles (PVA; Cook, Bloomington, Indiana) that were 150–250 μm in diameter.; (2) the TACE group (n = 15), in which embolization was performed using PVAs after the infusion of a suspension of 10-hydroxycamptothecin (1 mg/kg) and lipiodol (0.4 ml) (Lipiodol Ultra-Fluid; Guerbet, Paris, France) into the left hepatic artery; and (3) the control group (n = 9), which was administered a 0.4 ml distilled water infusion into the left hepatic artery. One-third of the animals in each group were euthanized 6 hours, 3 days, and 7 days after treatment. Liver tissues from the embolized and unembolized lobes were surgically removed and fixed in buffered 10% formalin for the immunohistological examinations.

### Magnetic Resonance Imaging

Magnetic resonance imaging (MRI) was performed 17–18 days after tumor implantation (and prior to embolization) using a 1.5 T unit (Magnetom Avanto; Siemens Medical Solutions, Erlangen, Germany). All animals underwent a T2-weighted turbo spin-echo (T2W TSE) sequence, with the following imaging parameters: TR/TE  = 3700/87 milliseconds, 4 mm slice thickness, 15% intersection gap, 168 Hz/pixel BW, 200*200 mm^2^ field of view, 320*320 matrix, turbo factor  = 11, and averages  = 2.

### Angiographic Procedures

Angiographic procedures were performed using a Siemens C-arm PowerMobile unit (Angiostar Plus, Siemens Medical Solutions, Munich, Germany) guidance. After surgically cutting the femoral artery, a 4 Fr vascular sheath (Terumo, Tokyo, Japan) was introduced, a 4-Fr Cobra visceral catheter (Terumo) was used to select the celiac axis, and digital subtraction angiography of the celiac axis was then performed. A 2.7 Fr coaxial catheter system (Terumo) was advanced superselectively into the left hepatic artery. Subsequent interventions were carried out after confirmation of vessel insertion and tumor staining using DSA, which was performed using 2 ml manual injections of full strength contrast media (Omnipaque 350; GE Healthcare, Shanghai, China).

For the TAE group, PVAs that had been reconstituted using contrast media were injected carefully into the left hepatic artery. The embolization endpoint was the complete stasis of antegrade blood flow. For the TACE group, embolization was performed using PVAs after the injection of the mixture of 10-hydroxycamptothecin and lipiodol. Treatment was always restricted to the left lobe during each session for both TAE and TACE. For the control animals, 0.4 ml of distilled water was injected instead of the PVAs.

### Blood Tests to Assess Liver Function

Blood samples were collected from all of the rabbits 6 hours, 3 days, and 7 days post-procedure. Plasma levels of alanine aminotransferase (ALT), aspartate aminotransferase (AST) and bilirubin were measured using standard enzymatic procedures.

### Immunohistochemistry

Liver tissue samples were fixed in 10% neutral buffered formalin and embedded in paraffin. Serial 5 μm thick liver tissue sections were deparaffinized and subjected to hematoxylin-eosin-safran, Sirius Red and α-SMA immunohistochemical staining.

For Sirius Red staining, the sections were stained with 1% Picro-Sirius Red (Sigma). After 1 hour of staining, the specimens were dehydrated, cleared, sealed and observed under a polarization microscope.

For immunohistochemical staining, alpha-smooth muscle actin (α-SMA) mouse monoclonal antibodies (Sigma, A2547, dilution: 1/100) were used as the primary antibodies. Immunolabeling was achieved using a Super Sensitive Link-Label Immunohistochemical Detection System (Dako, Glostrup, Denmark). First, the paraffin sections were dewaxed and washed with 3% hydrogen peroxide in methanol at room temperature for 10 minutes to block endogenous peroxidase activity. Then, the slides were incubated successively with the primary antibodies, as defined above, and incubated with a secondary anti-mouse immunoglobulin (Dako) for 30 min at room temperature. The binding reactions were visualized using a DAB (3-3-diaminobenzidine tetrahydrochloride) substrate. Finally, the tissue sections were counterstained with hematoxylin.

### Hepatocellular Necrosis

The severity of liver injury in the left lobes was graded using modified Suzuki's criteria by a researcher who was blinded to the type of procedure that had been performed. In this classification, sinusoidal congestion, hepatocyte necrosis, and ballooning degeneration are graded on a scale from 0 to 4. No necrosis, congestion, or centrilobular ballooning is assigned a score of 0, whereas severe congestion/ballooning and 60% or greater lobular necrosis is assigned a value of 4. Six microscopic fields for each sample were chosen randomly and evaluated at a magnification of 100X.

### Liver Fibrogenesis

Sirius Red stains collagen red on a pale yellow background under a bright-field microscope, whereas under a polarization microscope, collagen appears bright yellow-red and/or bright green [1], [13]. One section from each specimen, and five fields from each section, were selected randomly. The same measurement procedure was followed for each field, and the mean ratio was then calculated.

α-SMA staining was quantified using morphometric analyses. Labeled areas in the blinded specimens were measured at a video screen display magnification of 100X and expressed as the ratio of the labeled area per total analyzed field surface. The average of the ratios that were taken from 5 consecutive fields centered on a portal tract was used to generate a single ratio for each specimen.

### Statistical Analyses

Quantitative data are presented as the means±SD. Significance between groups was established by using the Kruskal-Wallis *H* and Mann-Whitney *U*-tests. Differences were considered to be significant if P<0.05. Statistical analyses were carried out using SPSS version 13.0 (SPSS, Chicago, IL).

## Results

### VX2 Tumor Model

A solitary VX2 tumor was successfully depicted in the left liver lobe of each animal using T2W TSE MRI. The tumor diameter varied from 1.0 to 1.6 cm. TAE and TACE were successfully performed in all animals, and no animals died unexpectedly during the experimental period.

### Liver Function

**Figure 1:**
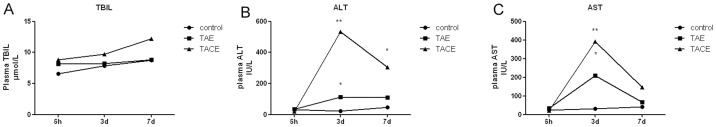
Postoperative changes in serum ALT, AST and bilirubin levels. (A and B) ALT and AST peaked on day 3 in the TAE and TACE groups. (C) Serum bilirubin levels increased slightly after all of the procedures, but these increases were not significant. *P<0.05 compared to the control group; **P<0.05 compared to the TAE and control groups.

All of the embolized animals showed marked elevations in ALT and AST values on day 3 to levels that were significantly higher than those in the control animals (P<0.05). The ALT and AST values in the animals in the TAE group peaked on day 3 and had decreased by day 7. The rabbits in the TACE group displayed higher levels of ALT and AST than those of the rabbits in the TAE group on day 3 (P<0.05), and higher levels of ALT than the control animals on day 7 (P = 0.034). The serum bilirubin values remained similar to control baseline in all of the animals throughout the experimental period [14] (Figures 1A, B, and C).

### Hepatic Necrosis

**Figure 2:**
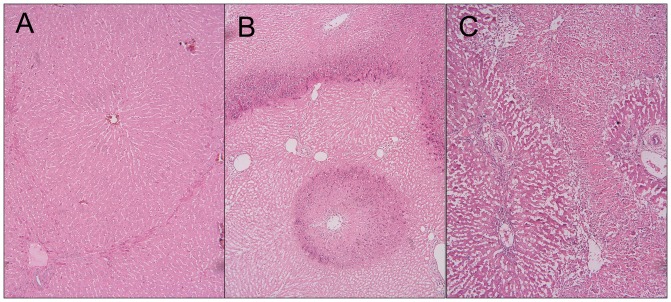
Histologic analyses on postoperative day 7. Hematoxylin and eosin stained livers adjacent to tumors from rabbits (100X). The lobular architecture in the control group was well preserved, and no edema, congestion, or centrilobular necrosis was observed (A). The TAE group displayed focal hepatocyte necrosis and the infiltration of inflammatory cells (B). The TACE group displayed severe sinusoidal congestion and hepatocyte necrosis. Diffuse infiltration of inflammatory cells (lymphocytes and granulocytes) and sinusoidal dilatation/atrophic trabeculae were observed (C).

Histopathologic examinations revealed no evidence of apparent hepatic necrosis in the control group. Focal necrosis with sinusoidal congestion and neutrophil accumulation was observed in the midzonal areas in the livers of the animals in the TAE group on days 3 and 7. In contrast, these changes were markedly increased in the animals in the TACE group at the corresponding times. The extent of hepatic parenchymal necrosis in the animals in the TACE group was significantly higher than that of the animals in the TAE group (Suzuki score of 2.33±1.29 versus 1.13±1.18, P = 0.001). In addition, the extent of hepatic parenchymal necrosis also differed markedly in the animals in the TACE group from the animals in the control group (Suzuki score of 2.33±1.29 versus 0.78±0.67, P = 0.006). The extent of hepatic parenchymal necrosis did not differ between the control and TAE groups [14] (Figures 2A, B, and C).

### HSC Activation and Liver Fibrogenesis Following TAE and TACE

**Figure 3:**
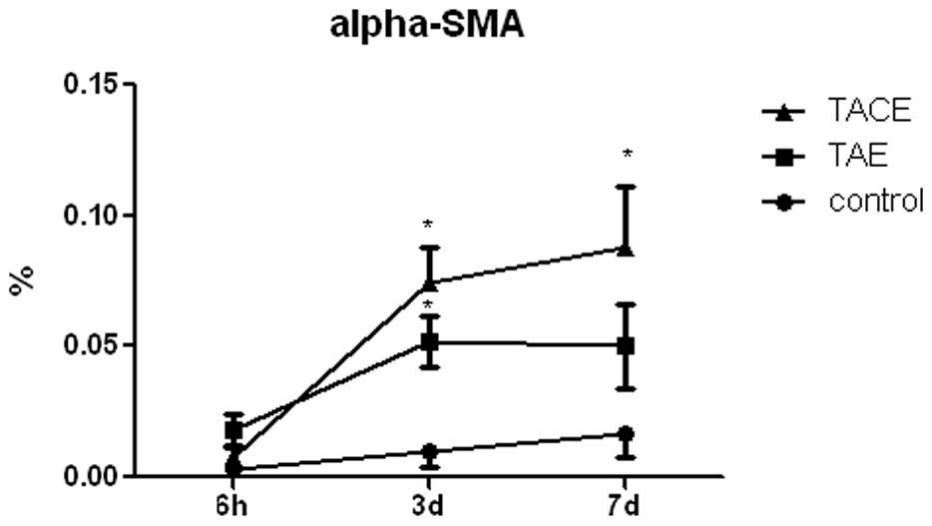
HSC activation following TAE and TACE at different time points after treatment. Areas that stained positive for α-SMA using immunohistochemistry were measured using a computerized image analysis system and expressed as a percentage of the total analyzed area. *P<0.05 vs. control.

**Figure 4:**
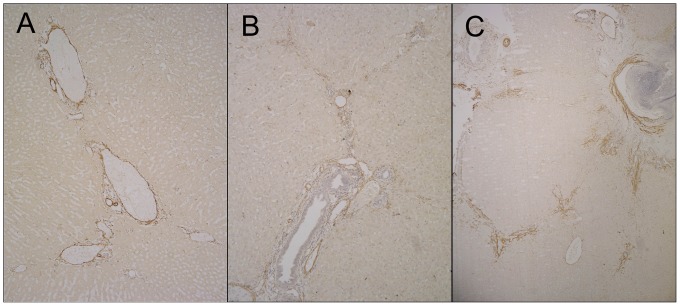
Immunohistochemical staining was used to determine HSC activation using α-SMA. Liver samples (embolized non-tumorous liver tissue) from the control, TAE and TACE groups 3 days after treatment. No positive α-SMA expression was observed in the control group, with the exception of the blood vessels (A). Significant positive α-SMA expression was mainly localized to the hepatic parenchymal cells near the portal tracts in the TAE group (B), and α-SMA expression was higher in the TACE group than in the TAE group (C).

Figure 3 shows α-SMA expression after treatment. α-SMA is expressed in activated HSCs and is not expressed in quiescent HSCs [15]. Therefore, we used α-SMA as a reliable marker for HSC activation to determine whether TAE and TACE induced HSC activation. No positive α-SMA expression was detected in the control group, or the unembolized liver tissues in the TAE and TACE groups, with the exception of the blood vessels (Figure 4A). No HSC activation was detected in the embolized liver tissues in the TAE and TACE groups 6 hours after treatment. However, on day 3 after TAE or TACE, significant positive α-SMA expression was mainly localized to the hepatic parenchymal cells near the portal tracts (0.052±0.010 and 0.074±0.014 *vs.* 0.010±0.006, *P*<0.05, as compared to the control group), and the expression of α-SMA was even higher in the TACE group than that in the control group by day 7 after treatment (0.088±0.023 *vs.* 0.017±0.009, P = 0.05) (Figures 4B and C).

**Figure 5:**
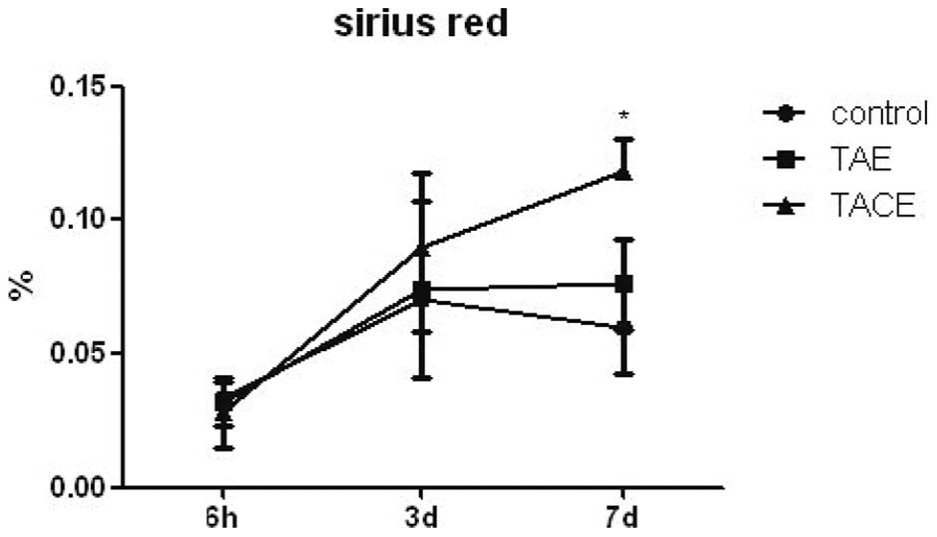
Liver fibrogenesis following TAE and TACE at different time points after treatment. Sirius Red stained areas were measured using a computerized image analysis system and expressed as a percentage of the total analyzed area. *P<0.05 vs. control.

**Figure 6:**
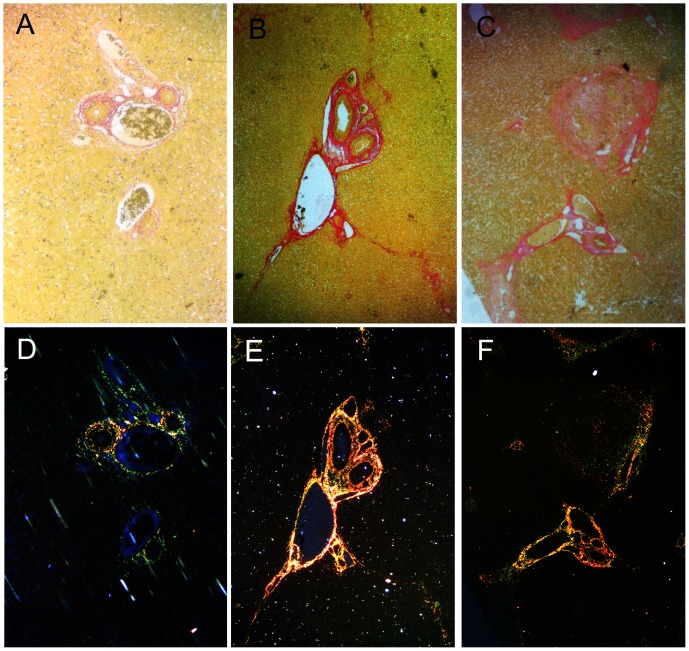
Picro-Sirius Red staining for liver fibrogenesis. Liver samples (embolized non-tumorous liver tissue) from the control, TAE and TACE groups 7 days after treatment. Picro-Sirius Red stains collagen red on a yellow background under a bright-field microscope, whereas under a polarization microscope, collagen appears bright yellow-red (mainly type I collagen), and/or bright green (mainly type III collagen). Seven days after treatment, Picro-Sirius Red staining was observed to be concentrated mainly around the portal tracts in the control group (A), and type I and III collagen fibrils were confined to the portal area (D). Seven days after embolization, liver fibrogenesis was detected in the TAE group (B). However, collagen fiber staining around the portal tracts was increased in the TACE group (C). In the TAE and TACE groups, dramatic increases in the levels of collagen fibrils, type I in particular, were observed (E and F).

Figure 5 shows liver fibrogenesis after treatment. Collagen fibers, as detected using Sirius Red staining, were observed to be concentrated mainly in the connective tissues around the portal tracts in the control group, but not in the hepatic parenchyma (Figure 6A). At hour 6 and day 3 after embolization, the embolized liver tissues did not display discriminatively hepatic parenchymal fibrogenesis. On day 7 after embolization, liver fibrogenesis was detected in the TAE group, but did not differ significantly from that in the control group (Figure 6B). However, collagen fiber staining near the portal tract and central veins was increased in the TACE group (0.118±0.012 *vs.* 0.060±0.017, P = 0.05) (Figure 6C).

Type III and I collagen fibrils were differentiated based on the different colors of interference and the intensity of collagen birefringence. Against a black background, thick yellow-red fibers were mainly type I collagen, while fine netlike green fibrils were mainly type III collagen. Type II and other tissues were not detected under the polarization microscope. In the control group, type I and III collagen fibrils were confined to the portal area (Figure 6D). In the TAE and TACE groups, alterations in the collagen fibril content, type I in particular, were dramatically observed. The bands of collagen fibrils extended from the portal areas to the hepatic sinuses and dissected liver lobules in these groups (Figures 6E and F).

## Discussion

Hepatic stellate cells (HSCs) are a remarkably diverse population of nonparenchymal cells in the liver. After any type of liver injury, HSCs undergo a transformation from cellular quiescence to activation and proliferate at the affected sites, preceding the production of extracellular matrix proteins and hepatic fibrogenesis [16]. Nonsuperselective embolization and chemotherapeutic agent infusion are well known to damage liver tissues (other than neoplastic nodules) and cause hepatocyte necrosis, bile ductal or portal vein endothelial damage, and inflammatory reactions. All of these factors may initiate or accelerate HSC activation and hepatic fibrogenesis.

To our knowledge, very few studies have shown that serum indicators of liver fibrosis, such as procollagen type- III, collagen type-IV, hyaluronate acid and laminin, are elevated after repeated TACE in hepatocellular carcinoma (HCC) patients [17]. Suou et al.[18] demonstrated marked increases in serum levels of the 7S fragment of type IV collagen, and a transient decrease in serum levels of the N-terminal propeptide of type III procollagen, following TAE. These studies reflected the accelerated synthesis and degradation of collagen in the liver that occurs after TAE/TACE. However, no histopathological evidence of liver fibrosis was mentioned. Kobayashi et al.[19] studied the influence of TACE on non-tumorous liver tissue, particularly the portal tract elements, and disclosed that a moderate to marked degree of peribiliary fibrosis frequently occurred in HCC patients who had a history of TACE compared to controls. However, this study failed to correlate these changes with underlying conditions, including therapies other than TACE, the amounts of embolizing particles, and anticancer drugs. Our study demonstrated that embolization, with or without anticancer drug injection, induced HSC activation and hepatic fibrogenesis in the embolized liver lobes, which participated in the wound healing response to liver damage that was caused by TAE and TACE.

We further investigated the influence of TAE, alone or combined with chemotherapeutic agents, on hepatocyte necrosis, HSC activation and fibrogenesis. In the rabbits in the TAE group, liver function, as assessed by measuring plasma AST, ALT, and bilirubin levels, was not markedly affected. In addition, liver parenchymal necrosis, as observed using histology, was mild, indicating that hepatic arteries that had been embolized with PVAs did not develop prominent hepatocellular damage. Moreover, HSC activation was self-limited and reversible. Hepatic fibrogenesis was not significantly increased during the observation period in the TAE group. In contrast, TACE lead to moderate to severe hepatocellular necrosis in the adjacent liver tissues, as revealed by significantly increased plasma AST, ALT, and bilirubin levels. Notably, HSC activation increased dramatically between 3 and 7 days in the TACE group as compared to the control group, and collagen levels were increased after 7 days as well. We speculated that the liver damage that was caused by TAE was associated with HSC activation and decreased as injury subsided. When TAE is combined with a mixture of anticancer drugs and ethiodized oil, it not only causes hepatic ischemia, but at the same time also increases the duration of contact between the poison and the non-tumorous liver tissues. Moreover, ethiodized oil may result in the obstruction of the peripheral vascular bed and the portal vein, thereby increasing liver damage [20], [21]. Our study provided evidence that the hepatocellular necrosis, HSC activation and fibrogenesis that were induced by TACE were more significant and persistent than those induced by TAE, which may increase the risk of hepatic parenchymal fibrosis.

Two key points that helped us to obtain the results described in this study should be addressed. First, the left hepatic artery was selectively catheterized, and the embolization endpoint chosen in our study was complete stasis of antegrade blood flow, which may have induced hypoxia not only in the tumor, but also in the left lobe. Second, a large quantity of α-SMA expressing cells and collagen fibers were detected in the liver tumors. Therefore, the histological pictures that were used for our analyses were centered on the portal tract area, and the tumor tissues were carefully excluded from the analyzed fields.

Although our experimental model has been generally accepted by many investigators to mimic TAE and TACE [22]–[24], the majority of cases of liver malignancy in humans, such as hepatocellular carcinoma, develop as a consequence of underlying liver disease (most commonly hepatic cirrhosis). Marked differences in the levels of activated HSCs exist between diseased human livers and normal rabbit livers. Therefore, the influence of TAE and TACE on HSC activation and fibrogenesis in diseased human livers may not be accurately reflected by our experiments. However, the data that we obtained using our experimental model provided useful information that may be applied to transcatheter treatments that are used for other hepatic tumor types, such as hepatic metastases, in which the background liver tissue is normal. We would also like to mention that the majority of clinical and experimental studies have demonstrated that the liver injury that was caused by TACE was self-limited, that HSC activation decreased over time, and that hepatic fibrogenesis may be reversible, none of which was evaluated in this study.

In summary, the results of the present study provided evidence that HSC activation and hepatic fibrogenesis may contribute to the progression of liver decompensation that is caused by TACE, which suggested that the beneficial effects of TACE in tumor necrosis may be counterbalanced by the deleterious effects of liver fibrosis. These results may shed light on the pathogenesis, treatment and prevention of liver damage that arises from TACE.
